# Use of Incentive Spirometry to Prevent Acute Chest Syndrome (ACS) in Patients With Sickle Cell Disease (SCD): A Systematic Review

**DOI:** 10.7759/cureus.91462

**Published:** 2025-09-02

**Authors:** Tasneem Elkanzi, Ghadeer MohamedTaha, Georgia Papageorgiou

**Affiliations:** 1 Internal Medicine, Northampton General Hospital, Northampton, GBR; 2 Internal Medicine, James Cook University Hospital, Middlesbrough, GBR; 3 Haematology, Northampton General Hospital, Northampton, GBR

**Keywords:** acute chest syndrome (acs), incentive spirometer, positive expiratory pressure, sickle cell anemia, vaso-occlusive crisis

## Abstract

Sickle cell disease (SCD) is a genetic hematological disorder that causes the production of sickle-shaped red blood cells. These abnormal cells reduce the oxygen-carrying ability around the body and obstruct blood flow, potentially resulting in devastating complications such as acute chest syndrome (ACS).

The objective of this systematic review is to assess whether incentive spirometry is effective in reducing the incidence of ACS in patients with SCD. This review searched databases, including the Cochrane Central Register of Controlled Trials (CENTRAL), MEDLINE, Embase, ClinicalTrials.gov, and the World Health Organization (WHO) International Clinical Trials Registry Platform (ICTRP) up to August 17, 2025.

Randomized controlled trials (RCTs) that used incentive spirometry in patients with SCD were included in the review. Inclusion criteria for this review include patients diagnosed with SCD who have received incentive spirometry, with outcomes compared against standard prevention for ACS or other alternative interventions aimed at preventing or managing lung pathology. Studies must report on at least one of the following outcomes: incidence of ACS, pulmonary function, hospital stay duration, hospitalization rates, or adverse effects.

This study found that three RCTs (29 patients, 38 patients, and 20 patients with a total of 124 hospitalizations) were included in this review. Two studies compared incentive spirometry to standard care, while only one compared incentive spirometry to positive expiratory pressure (PEP). A meta-analysis was conducted between two studies, with one trial suggesting that incentive spirometry successfully reduced the incidence of ACS and the other trial suggesting that it could not successfully reduce it. A meta-analysis of both studies found that incentive spirometry did not successfully reduce the incidence of ACS in patients with SCD (RR=0.51; 95% CI (0.21, 1.33)). The quality of this evidence was very low, due to the wide confidence interval, high risk of bias, and substantial heterogeneity.

This review concluded that incentive spirometry could not successfully reduce the incidence of ACS in patients with SCD. Limitations of the studies used included small sample sizes and heterogeneity between study populations (i.e., children vs. adults). Therefore, further research is required to assess this, including larger, well-designed RCTs to be conducted focusing on core outcome sets (COS).

## Introduction and background

Description of sickle cell disease (SCD)

SCD is a genetic blood disorder characterized by the production of abnormal hemoglobin, which causes red blood cells to adopt a rigid, sickle shape. The disease arises from a genetic mutation where valine replaces glutamic acid in the β-globin chain, leading to red blood cell deformities under low oxygen conditions. These deformities impair circulation and contribute to systemic complications [1]. This abnormality leads to increased blood viscosity, anemia due to premature cell breakdown, and the blockage of small blood vessels. The resultant complications include organ damage, painful vaso-occlusive crises (VOC), and oxygen deprivation in tissues. VOC is the most common clinical manifestation and can escalate into severe acute complications like acute chest syndrome (ACS) [2,3]. Globally, the World Health Organization (WHO) has identified that the prevalence of SCD has risen, with an estimated 7.74 million people affected by 2021. In the United Kingdom, about 14,000 individuals live with SCD, representing one in every 4,600 people [4,5].

ACS in SCD

ACS is a life-threatening complication of SCD caused by the obstruction of pulmonary microcirculation, often triggered by infection, hypoventilation, or rib infarction. It manifests as new pulmonary infiltrates on imaging, accompanied by symptoms like fever, chest pain, coughing, and difficulty breathing. ACS can progress to severe outcomes such as acute respiratory distress syndrome (ARDS), pulmonary infarction, fibrosis, and hypertension, significantly compromising lung function and quality of life. Recurrent ACS episodes exacerbate long-term pulmonary damage and mortality risks, underscoring the importance of prevention [6,7]. The management of ACS involves treating underlying causes and symptoms through interventions such as oxygen therapy, antibiotics, blood transfusions, pain management, and respiratory support techniques like incentive spirometry [8]. Preventative strategies include identifying and treating underlying risk factors with treatments such as hydroxyurea therapy and blood transfusions, along with incentive spirometry, which aims to reduce the incidence of ACS and its associated complications [7,9].

Introduction to incentive spirometry

**Figure 1:**
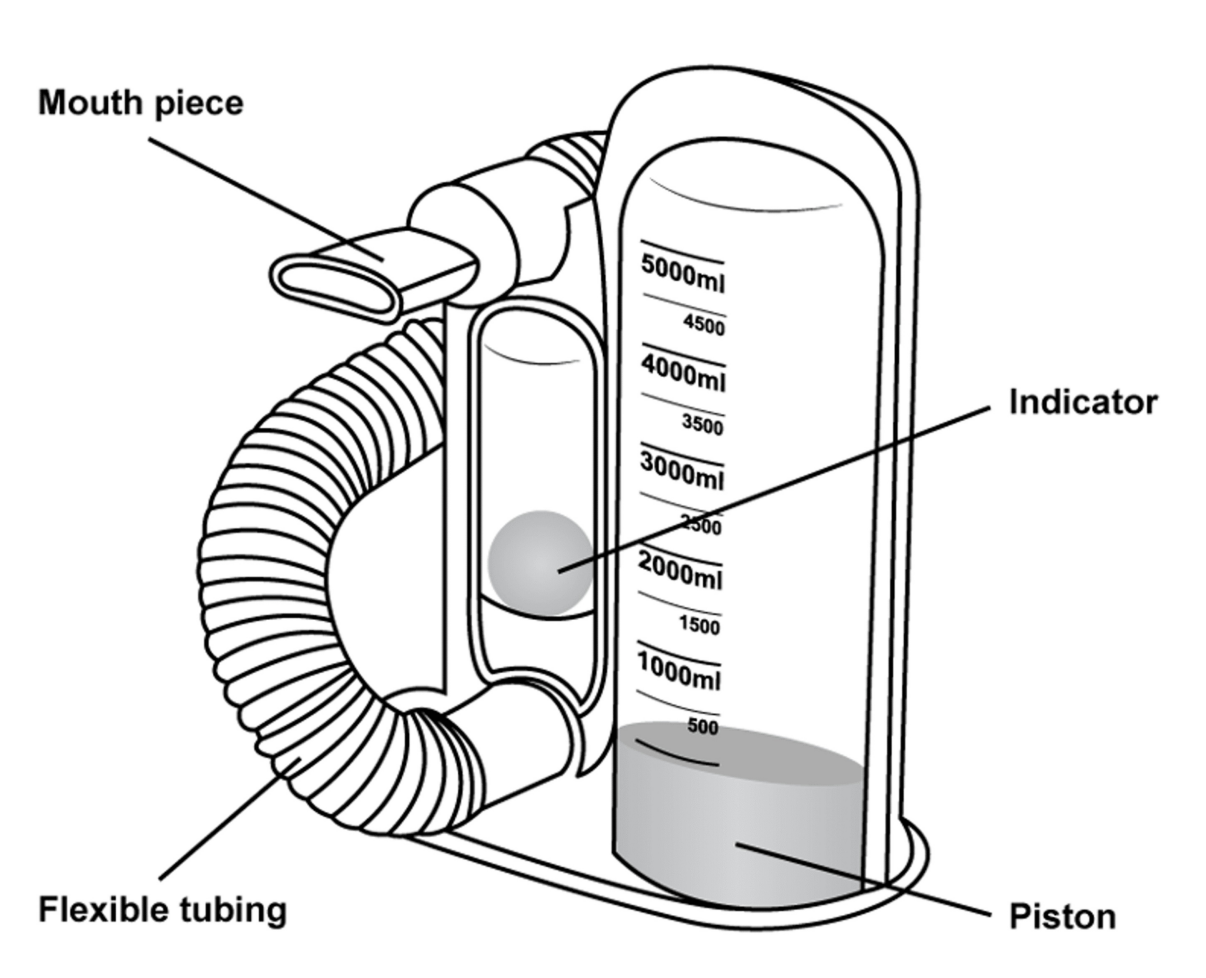
Volume-oriented incentive spirometer This illustration was designed by Tasneem Elkanzi to aid in the reader's visualization of an incentive spirometer.

Incentive spirometry is a non-invasive device designed to promote deep breathing and optimize respiratory function. The device provides visual feedback as patients perform sustained maximal inspiratory efforts, helping to prevent pulmonary complications such as atelectasis (Figure 1). It is commonly used in patients with respiratory diseases or those recovering from surgery. In the context of SCD, incentive spirometry aids in mitigating the risk of ACS by preventing hypoventilation and promoting lung expansion [10,11].

Importance of preventative measures

Preventing ACS is critical due to its high morbidity and mortality rates among SCD patients. ACS is the leading cause of death in SCD, contributing to 50% of fatalities in a UK descriptive study [12]. Effective prevention not only reduces hospitalizations but also helps to preserve lung function and reduce the risk of chronic lung disease. Incentive spirometers are advantageous because they are cost-effective and portable and encourage patient engagement. Moreover, their use has been suggested to reduce ACS-related hospitalizations, potentially leading to significant healthcare costs [13].

Rationale for a systematic review

Despite the potential benefits of incentive spirometry, existing evidence on its effectiveness in preventing ACS remains inconclusive due to the lack of available literature. A systematic review is necessary to synthesize data from randomized controlled trials (RCTs) and evaluate its efficacy in reducing ACS incidence and improving outcomes like hospital stay duration and pulmonary function. By aggregating findings, the review aims to identify patterns and provide a clearer understanding of the intervention's role in SCD management while also assessing potential adverse effects [14]. Overall, this review seeks to validate the effectiveness of incentive spirometry as a preventative measure for ACS, offering insights that could enhance patient care and inform clinical guidelines.

The abstract for this review was presented at the 2025 British Society of Haematology Conference on April 28, 2025.

## Review

Methods

Research Question Using the PICO Framework

The research question guiding this systematic review is as follows: "Does the use of incentive spirometry reduce the incidence of ACS in patients with SCD compared to standard care or other respiratory interventions?"

PICO Framework

Population: The population comprised individuals with SCD.

Intervention: The study used incentive spirometry.

Comparison: Standard sickle cell/vaso-occlusive treatment (e.g., oxygen therapy, pain relief, IV fluids, hydroxyurea, blood transfusions) and alternative respiratory therapies (e.g., continuous positive airway pressure (CPAP), non-invasive ventilation) were compared.

Outcomes: The study has two outcomes: for the primary outcome, the incidence of ACS and for the secondary outcome, pulmonary function improvement, hospitalization rates, hospital stay duration, and adverse effects.

Types of Studies

RCTs and quasi-RCTs were eligible for inclusion.

Types of Participants

The review considered children (≥3 years) and adults with all SCD genotypes, following evidence that children aged 3+ can use incentive spirometry effectively under supervision [15-17].

Types of Intervention

Eligible studies examined incentive spirometry for reducing the incidence of ACS in SCD patients, comparing its effects to standard care or alternative respiratory therapies.

Outcome Measures

The primary outcome is the incidence of ACS; this can be defined as the number of new ACS events during the study's period. This is measured through clinical diagnosis and criteria. This review will accept the identified trial authors' definition of ACS, given that it is supported by evidence.

The secondary outcomes are the following: (1) hospitalization rates (admissions per year), (2) hospital stay duration (days), (3) pulmonary function tests (e.g., FEV1, inspiratory capacity), and (4) adverse effects of incentive spirometry, such as atelectasis formation or chest infections if the device is used improperly and events reported by patients or healthcare providers and recorded by healthcare providers.

Search Methods for Identifying Studies

Comprehensive searches of published and unpublished studies were conducted up to August 17, 2025, without language or date restrictions, minimizing publication bias.

Databases

This review searched databases, including the Cochrane Central Register of Controlled Trials (CENTRAL), MEDLINE (via PubMed), Embase (via Ovid), ClinicalTrials.gov, and the WHO International Clinical Trials Registry Platform (ICTRP) (see Appendix 1).

Data collection and analysis

Selection of Studies

Two reviewers independently screened titles, abstracts, and full texts to identify eligible studies using the Rayyan software (Rayyan Systems Inc., Cambridge, Massachusetts, United States). Discrepancies were resolved by consensus or a third reviewer.

Data Extraction

Data were extracted using a standardized form capturing study characteristics, patient demographics, interventions, outcomes, and more (see Appendix 2). This process was independently validated by a second reviewer. 

Risk of Bias Assessment

Risk of bias in RCTs was evaluated using the Cochrane Risk of Bias 2 (ROB2) tool, assessing five domains (e.g., randomization, outcome measurement). Discrepancies were resolved through discussion, and trial authors were contacted to clarify incomplete data.

Measures of Treatment Effect

Binary outcomes (e.g., ACS incidence) were expressed as risk ratios (RR) with 95% confidence intervals (95% CI), while continuous outcomes (e.g., hospital stay) were presented as mean differences (MD) or standardized mean differences (SMD) with 95% CI.

Assessment of Heterogeneity

Heterogeneity was assessed using the chi-squared test (p<0.1) and I² statistic, categorized as low, moderate, substantial, or considerable. Potential sources of heterogeneity were explored.

Data Synthesis

Meta-analysis followed Cochrane guidelines. Fixed-effects models were used for homogeneous data, while random-effects models were applied for heterogeneity exceeding 30% (I²). For non-meta-analytic synthesis, the Synthesis without Meta-analysis (SWiM) framework was employed.

Reporting Certainty of Evidence

This review employed the Grading of Recommendations Assessment, Development and Evaluation (GRADE) approach to evaluate the quality and certainty of evidence, categorizing it into four levels: high, moderate, low, and very low. The assessment considered five key domains, namely, risk of bias, indirectness, imprecision, inconsistency, and publication bias, and assigned low certainty to quasi-RCTs unless large, unbiased effects were observed. A summary of findings table was also included to present key outcomes, intervention effects, and overall GRADE assessments [18].

Results of the search

**Figure 2:**
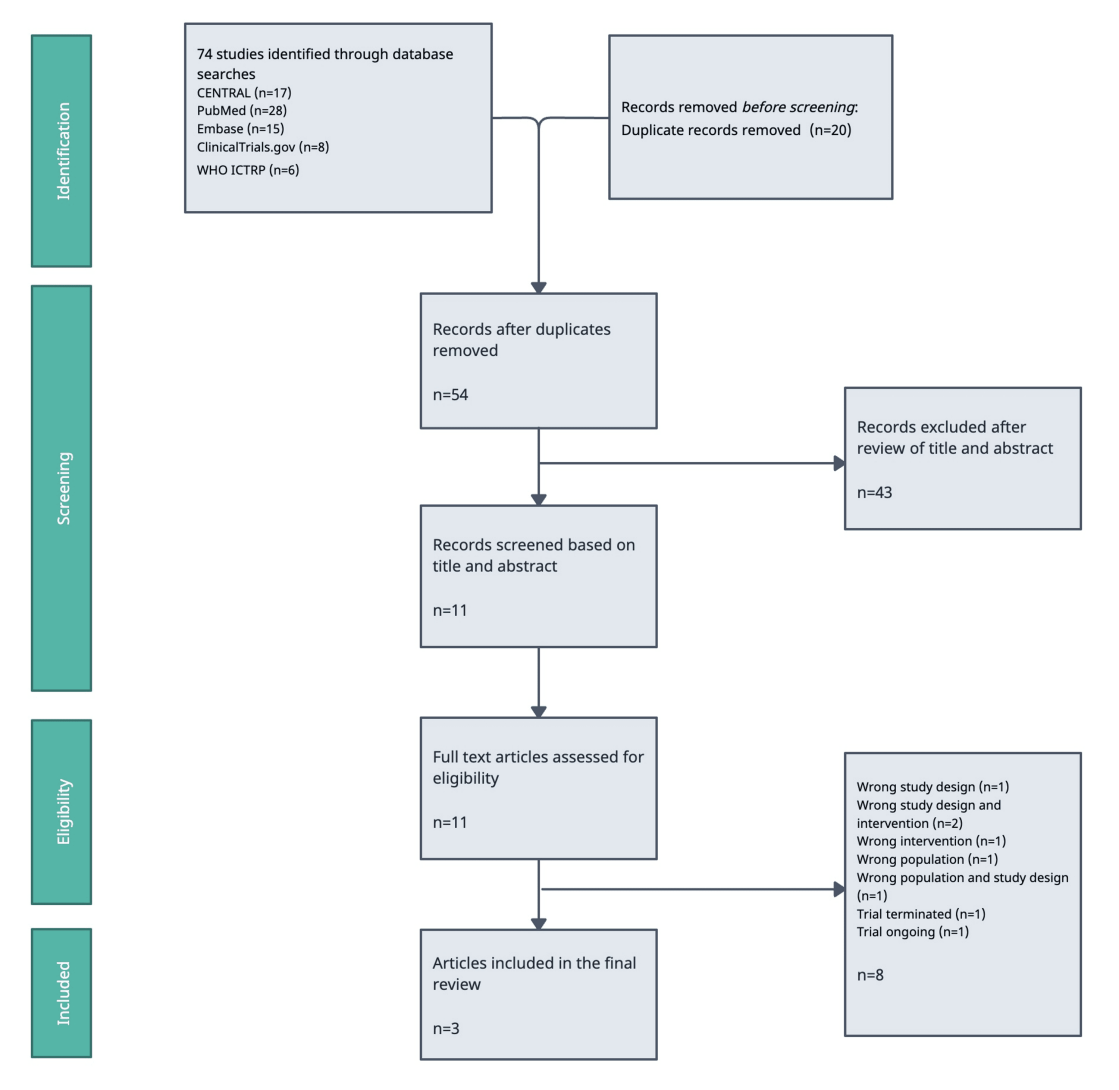
A four-stage PRISMA 2020 flow diagram CENTRAL: Cochrane Central Register of Controlled Trials; WHO ICTRP: World Health Organization International Clinical Trials Registry Platform; PRISMA: Preferred Reporting Items for Systematic Reviews and Meta-Analyses

A total of 74 studies were initially identified through database searches. After removing 20 duplicates using EndNote (Clarivate, London, United Kingdom), a citation management software, 54 studies remained. Title and abstract screening excluded 43 studies that did not meet the inclusion criteria. Of the remaining 11 studies, a further screening excluded eight after a full-text review, leaving three studies eligible for inclusion in the final review. Figure 2 shows the Preferred Reporting Items for Systematic Reviews and Meta-Analyses (PRISMA) flowchart [19].

Included studies

The review included three studies that met the eligibility criteria: Bellet et al. [20], Hsu et al. [21], and van Tuijn et al. [22]. These studies investigated the use of incentive spirometry in SCD for the prevention of ACS. Bellet et al. [20] and van Tuijn et al. [22] compared incentive spirometry to standard care, while Hsu et al. [21] compared incentive spirometry to positive expiratory pressure (PEP). See Appendix 3 for the characteristics of the included studies.

Trial design and setting

Bellet et al. [20] conducted a prospective RCT at a US hospital, treating each hospitalization as an independent event, which allowed for within-subject randomization. This design improved statistical power but introduced potential biases, such as carryover effects or variability in individual responses. Similarly, van Tuijn et al. [22] conducted a prospective RCT in two hospitals in the Netherlands, although it was unclear whether hospitalizations were treated as independent events. Hsu et al. [21], in contrast, employed a pilot RCT design at a single hospital, randomizing participants only once. This approach assessed the feasibility of comparing incentive spirometry and PEP and determining whether larger trials were warranted.

Interventions

All three studies compared incentive spirometry to alternative treatments for patients hospitalized with SCD. Bellet et al. [20] and van Tuijn et al. [22] compared incentive spirometry to standard care, which consisted of pain management and hydration following local protocols for managing VOC and ACS risk. Blood transfusions, antibiotics, and oxygen therapy were administered based on the attending physician's clinical judgment. Hsu et al. [21] compared incentive spirometry to PEP. In each study, participants in both intervention and control groups received standardized SCD/ACS care.

Outcomes

This systematic review evaluates whether incentive spirometry reduces the incidence of ACS in patients with SCD. Three studies were analyzed, each with distinct methodologies and objectives. Bellet et al. [20] examined pulmonary complications during hospitalization of SCD patients with chest or back pain, focusing on atelectasis and pulmonary infiltrates, which were often associated with ACS symptoms like fever and oxygen desaturation. The study also evaluated the impact of incentive spirometry on pulmonary function and hospital stay length, comparing outcomes between spirometry and non-spirometry groups. van Tuijn et al. [22] assessed the effectiveness of incentive spirometry in preventing ACS during VOC in hospitalized adults with SCD, focusing on differences in ACS incidence between standard care and incentive spirometry groups and exploring clinical and laboratory traits linked to ACS risk. Hsu et al. [21] analyzed patient satisfaction with incentive spirometry using a Likert scale and measured its impact on hospital stay length and ACS progression, defining ACS by new chest infiltrates and respiratory symptoms. The study also compared the efficacy of incentive spirometry to that of PEP devices.

Overall, the studies collectively explored incentive spirometry's role in preventing or mitigating ACS in SCD patients, with outcomes including incidence of ACS, hospital stay duration, pulmonary function, patient satisfaction, and related clinical traits. While each study approached the topic with unique metrics and methodologies, they highlight the potential value of incentive spirometry in managing pulmonary complications in this patient population.

Risk of bias in included studies

**Figure 3:**
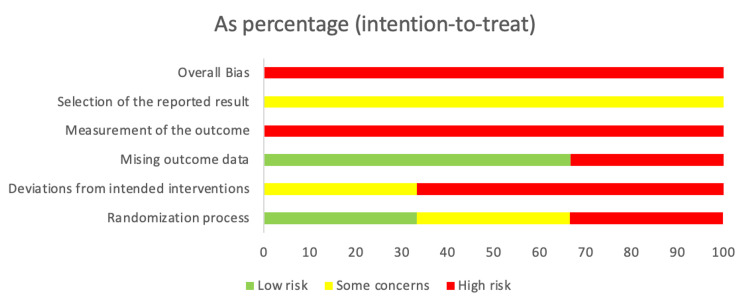
Risk of bias table: review authors' judgment about the included studies

**Figure 4:**
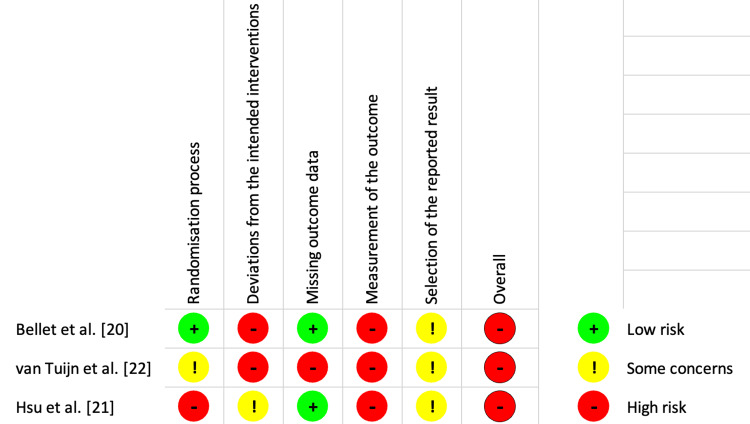
Risk of bias table: review authors' judgment about the included studies

Figures 3-4 represent the risk of bias analysis. All studies had high risks of bias, particularly from imprecision and study design limitations. This is due to methodological flaws such as inadequate or unclear randomization processes and a lack of allocation concealment. Outcome measurement was often subjective or potentially inconsistent across groups, raising concerns about detection bias. Small sample sizes in all studies contributed to imprecision and limited the ability to detect clinically relevant differences. Additionally, the absence of pre-registered protocols made it difficult to assess the completeness and reliability of the reported outcomes.

Effects of intervention

The quality of evidence has been assessed and graded for the outcomes stated in Tables 1-2. The included trials assess the effect of incentive spirometry vs. standard care and the effect of incentive spirometry vs. PEP in preventing ACS in patients with SCD.

**Table 1:** Summary of findings 1: IS versus standard treatment in patients with sickle cell disease who are at risk of developing ACS *Represents the RR or mean difference comparing PEP to IS, with a 95% CI **The absolute difference in risk between the control and intervention groups per 1000 patients GRADE Working Group grades of evidence: High: This study is very confident that the true effect lies close to that of the estimate of the effect. Moderate: This study is moderately confident in the effect estimate; the true estimate is likely to lie close to the effect estimate; however, there is a possibility that there is a significant difference. Low: This study is confident that effect estimate is limited. There may be a significant difference between the true effect and the estimate of the effect. Very low: This study has very little confidence in the effect estimate. There is a likely substantial difference between the true effect and the estimate of the effect. ^1^Downgraded for risk of bias ^2^Downgraded for imprecision ^3^Downgraded for inconsistency SC: standard care; IS: incentive spirometry; GRADE: Grading of Recommendations Assessment, Development and Evaluation; ACS: acute chest syndrome; RR: risk ratio; RCT: randomized controlled trial; CI: confidence interval; PEP: positive expiratory pressure

Outcomes	Anticipated absolute effect* (95% CI) SC vs. IS	Relative effect** (95% CI)	No. of participants/hospitalizations	Quality of the evidence (GRADE)	Comments
Incidence of ACS	SC: 275 per 1000; IS: 140 per 1000 (55-365 per 1000)	RR=0.51 (0.20, 1.33)	104 hospitalizations (2 RCTs)	⊕⊝⊝⊝ very low^1,2,3^	High risk of bias, wide CI, may have substantial heterogeneity
Hospitalization rates	SC: 275 per 1000; IS: 217 per 1000 (71-651 per 1000)	RR=0.79 (0.26, 2.37)	66 hospitalizations (1 RCT)	⊕⊕⊝⊝ low^1,2^	High risk of bias, wide CI
Length of hospital stay	Mean difference: -0.90 days (-2.41 to 0.61 days)	-	38 hospitalizations (1 RCT)	⊕⊕⊝⊝ low^1,2^	High risk of bias, wide CI
Lung function parameters	-	-	(0 RCT)	-	Limited data on lung function changes in both groups
Adverse effects	-	-	(0 RCT)	-	Lack of specific data on adverse effects

**Table 2:** Summary of findings 2: IS versus PEP in patients with sickle cell disease who are at risk of developing ACS *Represents the risk ratio or mean difference comparing PEP to IS, with a 95% CI **Expected outcomes per 1,000 patients, assuming the control (standard treatment) rate and the effect of the intervention (IS) GRADE Working Group grades of evidence: High: This study is very confident that the true effect lies close to that of the estimate of the effect. Moderate: This study is moderately confident in the effect estimate; the true estimate is likely to lie close to the effect estimate; however, there is a possibility that there is a significant difference. Low: This study is confident that effect estimate is limited. There may be a significant difference between the true effect and the estimate of the effect. Very low: This study has very little confidence in the effect estimate. There is a likely substantial difference between the true effect and the estimate of the effect. ^1^Downgraded for risk of bias ^2^Downgraded for imprecision GRADE: Grading of Recommendations Assessment, Development and Evaluation; ACS: acute chest syndrome; RCT: randomized controlled trial; CI: confidence interval; IS: incentive spirometry; PEP: positive expiratory pressure

Outcomes	Anticipated absolute effect* (95% CI)	Relative effect** (95% CI)	No. of participants	Quality of the evidence (GRADE)	Comments
Incidence of ACS	Neither participant in either group developed ACS	Neither participant in either group developed ACS	20 (1 RCT)	-	Neither participant in either group developed ACS
Hospitalization rates	-	-	(0 RCT)	-	No data available
Length of hospital stay	Mean difference: 0.70 days (-1.55 to 2.95 days)	-	20 (1 RCT)	⊕⊕⊝⊝ low^1,2^	Risk of bias, wide CI, small sample size
Lung function parameters	-	-	(0 RCT)	-	No data available
Adverse effects	-	-	(0 RCT)	-	No reported adverse effects identified

Incentive spirometry vs. standard care

ACS Incidence

**Figure 5:**
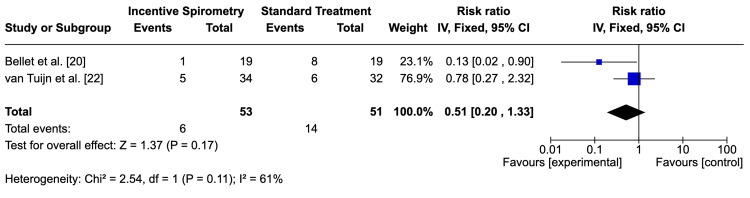
Meta-analysis for the incidence of ACS Meta-analysis for the incidence of ACS in the studies by Bellet et al. [20] and van Tuijn et al. [22] ACS: acute chest syndrome

Bellet et al. [20] reported an 87% reduction in ACS risk in the incentive spirometry group compared to standard care (RR=0.13; 95% CI: 0.02-0.90), indicating statistical significance. However, van Tuijn et al. [22] found no significant difference (RR=0.78; 95% CI: 0.27-2.32). The pooled analysis (RR=0.51; 95% CI: 0.27-2.32) suggested a trend favoring incentive spirometry but with considerable uncertainty (Figure 5). The GRADE assessment rated the evidence as very low due to bias, imprecision, and heterogeneity between studies (Table 1).

Hospitalization Rates

**Figure 6:**
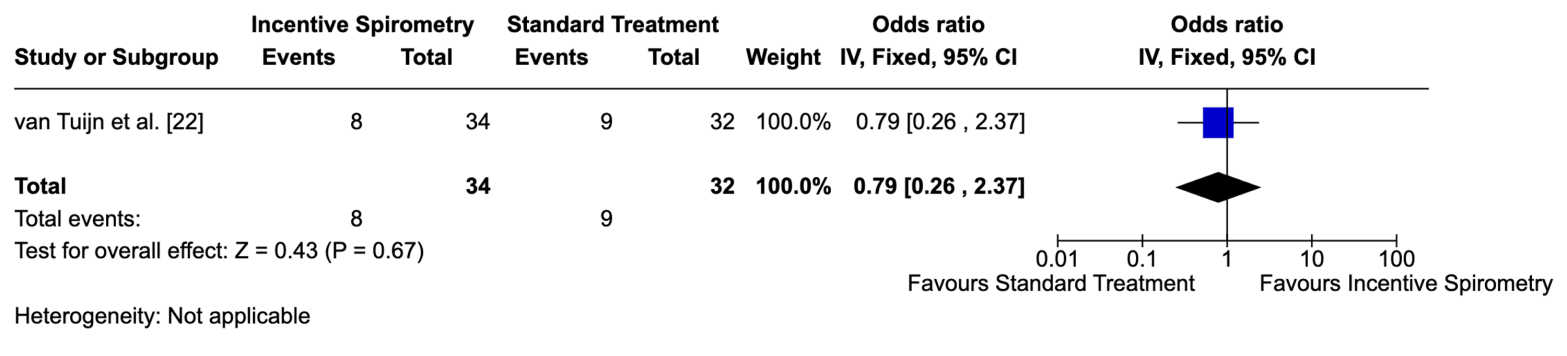
Relative risk of hospitalization rates Relative risk of hospitalization rates in the study by van Tuijn et al. [22]

Bellet et al. [20] did not analyze repeat hospitalizations. In contrast, van Tuijn et al. [22] reported slightly fewer repeat hospitalizations in the incentive spirometry group, but the wide confidence interval (RR=0.79; 95% CI: 0.26-2.37) suggested inconclusive evidence (Figure 6). This outcome was graded as low-quality evidence due to imprecision and high risk of bias (Table 1).

Length of Hospital Stay

**Figure 7:**
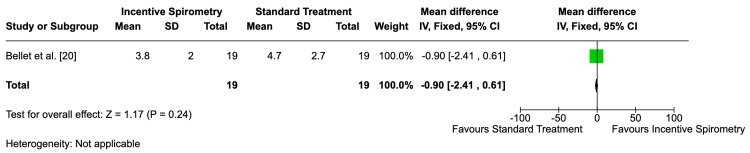
Mean difference of length of hospital stay Mean difference of length of hospital stay in the study by Bellet et al. [20]

Bellet et al. [20] found a mean reduction of 0.90 days in the incentive spirometry group compared to standard care (95% CI: -2.41 to 0.61), indicating uncertainty (Figure 7). Data from van Tuijn et al. [22] were insufficient for analysis. The GRADE assessment for Bellet et al.'s [20] findings was low due to a high risk of bias and imprecision (Table 1).

Adverse Effects

Both Bellet et al. [20] and van Tuijn et al. [22] reported no significant adverse effects associated with incentive spirometry. Narcotic use and mortality rates were comparable across groups, and no deaths were reported (Table 1).

Incentive spirometry vs. PEP

**Figure 8:**
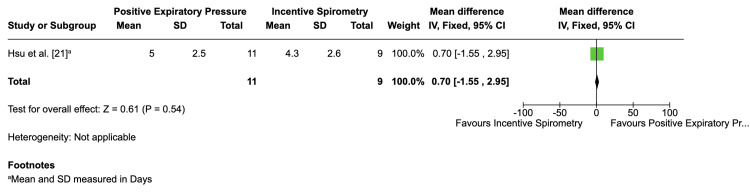
Mean difference of length of hospital stay Mean difference of length of hospital stay in the study by Hsu et al. [21]

Hsu et al. [21] compared incentive spirometry and PEP but reported no cases of ACS in either group. Due to the absence of ACS cases, no conclusion could be drawn regarding the relative effectiveness of incentive spirometry compared to PEP in preventing ACS. The study noted no adverse effects or dropouts, indicating both interventions were safe. The length of hospital stay was 0.70 days longer in the PEP group, but the wide confidence interval (-1.55 to 2.95) precluded definitive conclusions (Figure 8). Lung function parameters were not directly assessed, limiting the study's findings. The GRADE assessment for this trial was low due to its small sample size, high risk of bias, and imprecision (Table 2).

The evidence suggests incentive spirometry may reduce ACS risk in SCD patients, but significant uncertainty exists due to heterogeneity, small sample sizes, and methodological limitations. Adverse effects were minimal, and incentive spirometry appears safe. However, the overall quality of evidence is low to very low, emphasizing the need for larger, well-designed RCTs to confirm these findings. 

Discussion

ACS is a life-threatening complication of SCD that manifests as sudden chest pain, fever, and respiratory distress [23]. This review evaluated the effectiveness of incentive spirometry in reducing the incidence of ACS. Three studies met the inclusion criteria: two RCTs and one pilot RCT, encompassing 97 participants and 134 hospitalizations. These trials assessed the impact of incentive spirometry compared to standard treatment or PEP. Despite its potential, the findings from this review were inconclusive regarding the efficacy of incentive spirometry in significantly preventing ACS.

Two trials [20,22] compared incentive spirometry to standard SCD/VOC treatment. Bellet et al. [20] demonstrated a reduction in ACS progression among children and young adults, while van Tuijn et al. [22] found no significant benefits in adults. A meta-analysis of these studies revealed no statistically significant reduction in ACS incidence with incentive spirometry, partly due to moderate-to-substantial heterogeneity (61%) between the studies. The heterogeneity likely stemmed from differences in participant demographics, particularly age groups. Despite these differences, combining the studies allowed for broader insights into the intervention's potential across various populations. However, the quality of evidence was low, as assessed by the GRADE framework [19]. Issues such as a high risk of bias, small sample sizes, and wide confidence intervals limited the precision and reliability of the findings.

The third study compared incentive spirometry to PEP therapy but reported no cases of ACS in either group, preventing any relative risk calculation. This study found that incentive spirometry reduced hospital length of stay compared to PEP therapy, but the small sample size and high risk of bias limited the reliability of the findings. Across all studies, limited data were available regarding lung function, adverse effects, and hospitalization rates. Only one trial [22] reported on hospitalization rates, showing a lower risk of readmission in the intervention group but with considerable uncertainty due to a wide confidence interval.

Existing literature offers mixed evidence. For example, Ahmad et al. [24] found a significant reduction in ACS incidence among children with SCD using mandatory incentive spirometry, echoing the positive outcomes observed in Bellet et al. [20]. However, studies like Niazi et al. [25] suggest that effectiveness may vary by age group due to differences in ACS pathophysiology between children and adults. In children, ACS is most likely caused by viral and bacterial infections, whereas adults have a higher incidence of ACS secondary to fat embolism, thereby suggesting that the standard of care may differ in treating pediatric versus adult populations, with antimicrobial-directed treatment in pediatrics and treatment directed against the prevention of thromboembolism in adults [25]. This clinical heterogeneity in age groups between trials contributed to the downgrading of the GRADE score (Table 1).

One important aspect of this review was assessing the safety of incentive spirometry. Although no adverse effects were reported in the included studies, the literature suggests potential risks, such as atelectasis or respiratory infections, if the device is used improperly. However, these risks can be mitigated with proper education, supervision, and patient selection [26]. This underscores the importance of ensuring patients are adequately instructed on the correct use of incentive spirometry to maximize its benefits while minimizing potential harm.

The findings of this review underscore the need for further high-quality research. Future studies should prioritize large, well-designed RCTs focusing on specific age groups, particularly children, to reduce heterogeneity and assess age-related differences in effectiveness. The inclusion of core outcome sets (COS), as outlined by Tambor et al. [27], will ensure that trials measure clinically meaningful outcomes such as ACS incidence, quality of life, and hospitalization rates, improving comparability. This can be done with a patient-centered approach, involving stakeholders, who are individual people or a group of people who are affected by or can affect a research project's outcomes [28]. This aims to improve trial design, recruitment, and relevance throughout the research process. Some of the suggested COS for future ACS clinical trials include frequency of hospitalization, requirement of blood transfusion, ability to return to usual activity, health-related quality of life, and morbidity and mortality [27].

## Conclusions

This review could not confirm the efficacy of incentive spirometry in significantly reducing ACS incidence in patients with SCD. Although the evidence indicated uncertainty, it highlighted its potential to reduce hospital stays and identified critical gaps in the literature and the need for further research to clarify the role of incentive spirometry in ACS prevention. ACS prevention remains a crucial goal for improving outcomes in SCD patients, and future research, including larger, well-defined RCTs that focus on COS, should be conducted to refine the role of incentive spirometry in this context. Integrating COS and tailoring trials to specific populations will enhance the relevance and applicability of future findings, ultimately guiding evidence-based strategies for ACS prevention.

## Appendices

Appendix 1: search strategy

Cochrane Central Register of Controlled Trials (CENTRAL) Search Strategy

#1

(Sickle NEXT Cell NEXT Anemia* OR Sickle NEXT Cell NEXT Aneamia* OR Sickle NEXT Cell NEXT Anaemia* OR Hemoglobin NEXT S NEXT Disease* OR Sickle NEXT Cell NEXT Disease* OR Sickle NEXT Cell NEXT Disorder* OR Sickling NEXT Disorder NEXT Due NEXT to NEXT Hemoglobin NEXT S OR HbS NEXT Disease OR drepanocytemia OR drepanocytic NEXT anaemia OR drepanocytic NEXT anemia OR drepanocytosis OR haemoglobin NEXT SS OR haemoglobin NEXT SS NEXT disease OR Hb NEXT SS NEXT disease OR hemoglobin NEXT SS OR hemoglobin NEXT SS NEXT disease OR homozygous NEXT Hb NEXT S NEXT disease OR homozygous NEXT sickle NEXT cell NEXT anaemia OR homozygous NEXT sickle NEXT cell NEXT anemia OR homozygous NEXT sickle NEXT cell NEXT disease OR meniscocytosis OR sickle NEXT anaemia OR sickle NEXT anemia OR sickle NEXT cell NEXT anaemia OR sickle NEXT cell NEXT disease OR sickle NEXT cell NEXT disorder OR SS NEXT dis*):ti,ab,kw

#

(Acute NEXT Chest NEXT Syndrome* OR pulmonary NEXT complication* OR lung NEXT complication* OR respiratory NEXT complication* OR pulmonary NEXT adverse NEXT event* OR lung NEXT adverse NEXT event* OR respiratory NEXT adverse NEXT event* OR pulmonary NEXT dysfunction* OR lung NEXT dysfunction* OR respiratory NEXT dysfunction* OR pulmonary NEXT disorder* OR lung NEXT disorder* OR respiratory NEXT disorder* OR pulmonary NEXT condition* OR lung NEXT condition* OR respiratory NEXT condition*):ti,ab,kw

#3

incentive:ti,ab,kw AND (spiromet* OR spirograph* OR spirogram OR Bronchospiromet* OR bronchospirograph*):ti,ab,kw) OR (breathing NEXT exercise*):ti,ab,kw

#5

Search: #1 AND #3

#4

Search: #1 AND #2 AND #3

#5

Search: #1 AND #3

#6

Search: #4 OR #5

MEDLINE via PubMed Search Strategy

#1

Search: "Anemia, Sickle Cell"[Mesh] OR "Sickle Cell Anemia*"[tiab] OR "Sickle Cell Aneamia*"[tiab] OR "Sickle Cell Anaemia*"[tiab] OR "Hemoglobin S Disease*"[tiab] OR "Sickle Cell Disease*"[tiab] OR "Sickle Cell Disorder*"[tiab] OR "Sickling Disorder Due to Hemoglobin S"[tiab] OR "HbS Disease"[tiab] OR "drepanocytemia"[tiab] OR "drepanocytic anaemia"[tiab] OR "drepanocytic anemia"[tiab] OR "drepanocytosis"[tiab] OR "haemoglobin SS"[tiab] OR "haemoglobin SS disease"[tiab] OR "Hb SS disease"[tiab] OR "hemoglobin SS"[tiab] OR "hemoglobin SS disease"[tiab] OR "homozygous Hb S disease"[tiab] OR "homozygous sickle cell anaemia"[tiab] OR "homozygous sickle cell anemia"[tiab] OR "homozygous sickle cell disease"[tiab] OR "meniscocytosis"[tiab] OR "sickle anaemia"[tiab] OR "sickle anemia"[tiab] OR "sickle cell anaemia"[tiab] OR "sickle cell disease"[tiab] OR "sickle cell disorder"[tiab] OR "SS dis*"[tiab]

#2

Search: "Acute Chest Syndrome"[Mesh] OR "Acute Chest Syndrome*"[tiab] OR "pulmonary complication*"[tiab] OR "lung complication*"[tiab] OR "respiratory complication*"[tiab] OR "pulmonary adverse event*"[tiab] OR "lung adverse event*"[tiab] OR "respiratory adverse event*"[tiab] OR "pulmonary dysfunction*"[tiab] OR "lung dysfunction*"[tiab] OR "respiratory dysfunction*"[tiab] OR "pulmonary disorder*"[tiab] OR "lung disorder*"[tiab] OR "respiratory disorder*"[tiab] OR "pulmonary condition*"[tiab] OR "lung condition*"[tiab] OR "respiratory condition*"[tiab]

#3

Search: (incentive[tiab] AND ("Spirometry"[Mesh] OR spiromet*[tiab] OR spirograph*[tiab] OR spirogram[tiab] OR Bronchospiromet*[tiab] OR bronchospirograph*[tiab]) ) OR "breathing exercise*"[tiab]

#4

Search: #1 AND #3

#5

Search: #1 AND #2 AND #3

Embase via Ovid Search Strategy

#1

exp Anemia, Sickle Cell/

#2

Hemoglobin, Sickle/

#3

(h?emoglobin S or h?emoglobin SC or h?emoglobin SE or h?emoglobin SS or h?emoglobin C disease or h?emoglobin D disease or h?emoglobin E disease or Hb SC or HbSC or HbAS or HbSS or HbAC or Hb SE or Hb SS or Hb C disease or Hb D disease or Hb E disease or SC disease*).mp.

#4

(sickle cell* or sicklemia or sickled or sickling or meniscocyt* or drepanocyt*).mp.

#5

(SCD and sickle).mp.

#6

((Hb S or HbS or sickle) adj3 (disease* or thalass?emi*)).mp

#7

1 or 2 or 3 or 4 or 5 or 6

#8

exp Acute Chest Syndrome/

#9

exp Lung/

#10

(lung* or Pulmonary infarction or pulm*).mp.

#11

8 or 9 or 10

#12

exp spirometry/

#13

respiratory intervention*.mp.

#14

exp Respiratory Therapy/ or Respiratory Therapy.mp.

#15

incentive spirometry.mp.

#16

12 or 13 or 14 or 15

#17

7 and 11 and 16

World Health Organization (WHO) International Clinical Trials Registry Platform (ICTRP) Search Strategy

#1

Sickle AND spirometry

ClinicalTrials.gov Search Strategy

#1

Condition: Sickle Cell Anaemia

Intervention: Spirometry

Appendix 2

**Table 3:** Data extraction sheet

Data extraction sheet	
Study	Title:
Methods	Allocation method:
	Participant masking:
	Participant exclusion after randomization:
	Percentage of and cause for follow-up losses:
Participants	Sample size:
	Ethnic origin:
	Sex:
	Age:
	Inclusion criteria:
	Exclusion criteria:
Intervention	Intervention group:
	Duration:
	Frequency of usage:
Control	Comparison group:
	Duration:
	Frequency of usage:
Outcomes	Primary
	Incidence of acute chest syndrome:
	Secondary
	Hospitalization rates:
	Length of hospital stay:
	Lung function parameters:
	Incidence of adverse effects related to incentive spirometry:

Appendix 3

**Table 4:** Characteristics of the included studies (objectives in bold are those that align with this review) RCT: randomized controlled trial; ACS: acute chest syndrome; PEP: positive expiratory pressure; VOC: vaso-occlusive crises; SCD: sickle cell disease

Study	Bellet et al. [20]	van Tuijn et al. [22]	Hsu et al. [21]
Methods	Two-arm prospective RCT; 1 center, located in the United States	Two-arm prospective RCT; 1 center, located in the Netherlands	Two-arm pilot RCT; 1 center, located in the United States
Participants	29 patients were randomized with each hospitalization considered as a new event, totalling 38 hospitalizations	48 participants with a total of 66 hospitalizations with each hospitalization treated as a separate event	20 participants
	Inclusion criteria: Patients between the ages of eight and 21 with normal or unchanged chest radiographs on admission	Inclusion criteria: Adult (age not specified) sickle cell disease patients admitted to the hospital for acute chest or back pain above the diaphragm	Inclusion criteria: Patients with infiltrates on chest radiograph upon admission; those with cognitive delays; children below the age of six; however, the average age of children included in the trial was 12.3 years for PEP and 8.8 years for incentive spirometry. This may be due to a misclassification or misunderstanding during the enrolment process, leading to the inclusion of older children despite the stated exclusion criteria
	Exclusion criteria: Patients below the age of 7; those with significant comorbidities	Exclusion criteria: Pediatric patients (age not specified); pregnant women; patients presenting with ACS; those who received blood transfusion in the last 3 months	-
Interventions	Intervention group: Incentive spirometry with patients instructed to take 10 maximal inspirations using an incentive spirometer every two hours during the day and while awake at night until their chest pain subsided. In addition to standard medical treatment as stated below	Intervention group: Incentive spirometry in addition to standard care (stated below)	Intervention group: PEP
	Comparison group: Standard medical management, which included intravenous fluids, oxygen therapy, pain relief, and antibiotics when necessary	Comparison group: Standard treatment only including pain management and hydration according to local protocols. Furthermore, antibiotics, oxygen therapy, and blood transfusion were administered on a clinical need basis	Comparison group: Incentive spirometry
Outcomes	Primary: Incidence of pulmonary complications, specifically atelectasis or pulmonary infiltrates	Primary: To evaluate the efficacy of incentive spirometry in preventing ACS during hospitalization for VOC in adult SCD patients	Primary: To compare patient satisfaction with therapy and the length of hospital stay between the intervention and control groups
	Secondary: Incidence of thoracic bone infarction, duration of hospital stays, the need for additional interventions such as blood transfusions or increased analgesic use, improving inspiratory capacity, reducing the overall duration of chest pain experienced by patients	Secondary: To assess whether specific clinical or laboratory characteristics were associated with an increased risk of developing ACS	Secondary: To assess the progression to ACS, defined by new infiltrate on chest radiograph plus respiratory symptoms
